# 

**Published:** 1871-01

**Authors:** 


### INDEX.

**A** PAGE

Anus, Imperforate........ 15
Anatomy, Early History........ 40
Army, Surg. Gen. Report........41, 122
Alumni Meeting........48, 149
Arsenic in Phthisis........ 57
American Practitioner........126, 672
American Journal Obstetrics........127, 371
Anatomical, Bill........ 128
Abdominal Effusions, Relief........ 225
American Medical Association........255, 356
Aspermatism........ 264
Across the Continent........ 308
Alcohol, Exp. on Effects........ 490
Abdominal Typhus........ 585

**B**

Board of Health........ 45
Belladonna, Typhoid Fever........ 54
Bismuth, Infantile Diarrhœa........55, 318
Bliss & Sharp........ 252
Brain, Punct. Wound, Recovery........ 294
Bladder, Malposition........ 347
Brainard Medical Society........ 384
Blood, Corpuscle, Red........ 561
Bigelow, Medical Education........ 660
Bromo-Chloralum........ 673

Book Notices:
Galvano-Therapeutics—*Neftel*........ 36
Physics and Physiology of Spiritualism 37
Specific Med. and Spec. Medicines—*Scudder*........ 38
Transactions Am. Med. Association........ 39
Fractures and Dislocations—*Howe*........ 39
Satan in Society........108, 122, 251, 504
Dis. of Rectum—*Van Buren*........ 120
Books Received........121, 183, 302, 444, 647
Billroth's Surg. Pathol. and Therap........ 233
Medical Chemistry—*Rand*........ 238
Reflex Insanity in Women........ 240
Modern Therapeutics—*Naphey*........ 240
Legendary and Mythological Art........ 241
Tanner, Infancy and Childhood........ 440
A Physician's Counsel, etc.—*Taylor*........ 559
Nervous System—*Hammond*........ 561
Opium and the Op. Appetite........ 644
Obstet. and Gynæcol. Works—*Sir J. Y. Simpson*........ 645

**C**

Chloral, Hydrate....12, 51, 344, 353, 376, 377
Cœci Appendix, Foreign Body in, etc... 17
Cœcitis, etc........ 19, 75
Croup and Cubebs........ 53
Cancrum Oris, Heat........ 56
Carbolic Acid in Phthisis and Diphtheria 56
Carbolic Acid, Preparations........ 57
Cigarettes, Medicated........ 59
Croup and Lactic Acid........ 62
Cathartic Pills, Officinal........ 147

PAGE

Carbolic Acid, Poisoning by........ 192
Criticism, Verat. Virid., Scarlatina..208, 331
Combustion, Spontaneous........ 224
Carrion Flies........ 251
Correlation Phys. and Vital Forces..276, 367
Chicken Cun........ 308
Chloral, Large doses........ 315
Cinchona in Java........ 320
Croup, Bromide Potassium........ 351
Children's Diseases, Quinine........ 378
Children's Sickness and Diarrhœa........ 378
Circular to Alumni Ch. Med. College........ 383
Carbolic Acid in Diphtheria, Aff........ 385
Cancer Cells, Micros. Ap........ 391
Colitis, Subacute........ 409
Cranium, Fracture........ 469
Children, Massacre of the Innocents.. 500
Cundurango........ 503, 505
Cataract, Statistical Report........ 519
Cholera in Pregnancy and Childbirth........ 536
Correspondence, Home........ 542
Cholera, Asiatic........ 566
Children, Feeble Minded........ 576
Cholera Infantum........ 666
Calamity........ 670

**D**

Diphtheria, Treatment........ 54
Death, Diagnosis........ 56
Dyspepsia Cured by Milk........ 79
Devil, Uses of........ 248
Degeneration—*Hitchcock*........ 305
Diaphoresis, Therap. Value........ 349
Delirium Tremens........ 380
Doctors in U. S........ 447
Different Diagnosis Acute Phthisis and Typhoid Fever........ 487
DeWitt County Medical Society........ 511
Death, Legal and Actual........ 600

**E**

Erysipelas, Sulphite Soda........ 20
Exchanges, Notice of Several Non-Prof. 43
Electro-Therapeutics........ 92
Education, Medical, in England........ 176
Error, Organ-ic........ 251
Emboli—*Hay*........ 291
Epistolary........ 295
Eye, Intraocular Ossification........ 449
Ear, Physiology—Sound........ 465
Epilepsy, Syphilitic........ 549

**F**

Foundlings, Mortality........ 55
Foreign Correspondence........ 176
Femur, Fract., Non-Union, etc.—*Parkes* 338
Fœtus, Extr. Uter., Deliv. through Abdominal Walls........ 406
"Formula No. 2."........ 471

G

PAGE

Grape Sugar, Improved Test........... 52
Gastric Douche........................ 58
Gynæcological Journal, Boston......... 239
Gutta Rosacea......................... 267
Glue Resisting Moisture... ............ 366
Gaillard-Yandell, Imbroglio............ 446
Gynæcological Clinique R. M. C........ 527

H

Hospital, Cook County, Clinic..8, 94, 171, 254
Harelip, Operations—*Hotz*............. 88
Humerus, Fract. Anat. Neck............ 214
Humerus, Excis. Head—*Clark*......326, 462
Half Yearly Compendium.............. 371
Hepatic Abscess, etc., Successful Treat.. 412
Hypodermic Medication.... ......... 418
Hearing, Mechanism.................. 436
Harvard Univ. Medical School... ...... 447
Hæmatemesis, Transfusion............ 507
Homœopathy, Modern Tendency....... 608

I

Items, News and Gossip.............49, 188
India Rubber, White.................. 51
Infallibility........................ ... 52
Impotence Cured...................... 54
Intestinal Obstruction.......... ..129, 321
Intermittent, I K, in .................. 192
Inebriates, Report American Association 241
Illinois State Medical Society........313, 570
Intestine, Intern. Strang................ 314
Iodine Paint, New.................... 317
Intermittent, Treatment................ 355
Intestinal Concretion.................. 446
Iron, Elixirs containing................ 498

J

January, 1871, Editorial............... 44

L

Lithotomy............................. 62
Lady Doctor—"*Pome*,"............... 62
Labor, Preternatural.................. 135
Lying Made Easy..................... 371
Lens, Micros. Jour..................... 564
Library, Medical, in Chicago........... 568

M

Malpractice—Charge of Judge Thayer... 29
Malt Extract.......................... 47
Matter, Living and Dead.......... ... 193
Memoirs, Med. and Surg.—*Jones*....... 309
Morbid Specimen...................373, 570
Meat Prepared in Java................ 381
Milk, Abnormal, Nutrition............. 439
Military Tract, Medical Society........ 447
Michigan Mineral Springs............. 473

N

Neuralgia Mixture.................... 39
New Hampshire Medical Society, Trans. 42
New York Ear Dispensary............ 509
North Iowa Medical Society........... 510
Nose, Foreign Body in................ 526

O

Orbit, Cavernous Tumor, etc........... 1
Obituary—*H. F. Chesbrough*, *M.D.*.... 63
Opium in Metro Peritonitis......115, 220, 306

PAGE

Obituary — *E. A. Clark*, *Niemeyer*, *Scanland*, *Hooker*, *Noble*...... ..... 310
Orchitis, Treatment................... 369
Opium................. ............ 477
Obstetrical Forceps, Management....... 623

P

Pemphigus, Chronic................... 4
Palpitation, Diagnostic Value.......... 21
Puerperal Convulsions, Chloral in...... 58
Phosphate of Lime in Sweating........ 119
Pamphlets Received....123, 246, 304, 650, 667
Protection.......................... 127
Pepsine and its Incompatibles.......... 212
Peppermint Oil, Anæsthetic........... 315
Paraphymosis, Treatment.............. 368
Petroleum in Dry Rot................ 369
Parke, Jennings & Co................. 371
Placenta Prævia...................... 375
Poisons, Effect on different Animals.... 379
Pepsine and Medical Preparations...... 381
Prurient Prudes...................... 446
Perineum and Rectum, Laceration...... 525

Q

Quackery, Illustrations..............55, 124

R

Rogers, E. C., M.D., Marine Hospital... 125
Responsibility........................ 126
Rush Medical College, Com............ 149
Replies to Senex.......... ........608, 613
Richardson, J. G., Address............ 657
Rush Medical College, Session 1871 and 2, 669

S

Schoolmaster Abroad.................. 52
Soil and Disease...................... 59
Skin, Transplantation................. 219
Syphilis, Nervous........... ........ 257
Surgical Cases—*Clark*................ 288
Scleriasis—Scleroderma............ .. 493
Sympathetic Nerve, Chart............. 505
Sciatica, Treatment.................. 508
Senex............................... 542
Sponge Tents....... ................ 577
Surgical Clinic, Prof. Gunn............ 593
Seaverns, Joel, M.D., Address.......... 650

T

Trismus Nascentium.................. 14
Toothache.......................... 58
Transfusion...................203, 507, 595
Typhoid Fever, Cold Baths. .......... 230
Typhoid Fever, Strychnia.............. 341
Tetanus.................. ......... 379
Tin Foil Wrapping................... 380
Turkish Baths....................... 567

U

Uterine Dis., Instruments....80, 138, 267, 398
Uterus, Enlargement.................. 316
Ureters, Pulsation................ ..... 377

V

Veratrum Viride, Prac...... ....65, 208, 331
Vivisections, Prof. Freer............... 200
Vesico-Vaginal Fistula................ 454

W

Water and Fire, Therap. Agents....... 513

### Non-Professional Exchanges.

***The Scientific American***

Grows upon our affections constantly. We had the general idea, previously to being its regular readers, that it would be of little interest save to inventors and manufacturers Belonging to neither of these classes, nevertheless we find but few articles in its crowded columns and large space that do not interest us. It is the first paper picked up, and the last one laid down by patients waiting in the office. It is, briefly, exactly what it purports to be, "A Weekly Journal of Practical Information, Art, Science, Mechanics, Chemistry and Manufactures," profusely illustrated, and published by Munn & Co., 37 Park Row, New York.

***The American Chemist.***

A Monthly Journal of Theoretical, Analytical and Technical Chemistry. Edited by Chas. F. Chandler, Ph. D., and W. H. Chandler. Published by William Baldwin & Co., 434 Broome street, New York.

We have inadvertently omitted to announce this paper, which the enterprising proprietors now publish as an original journal in lieu of reprinting, as heretofore, an English copy.

Whoever would keep pace with the beautiful science of chemistry in all its departments, can find in this journal just what he wants. Each No. contains forty pages or more of reading matter in quarto form, with "cut leaves." $5 a year; 50 cents single No.; specimen copies 35 cents.

***Harper's New Monthly Magazine***

Enters upon its Forty-Second Volume with the January No. of 1871. Its table of contents is, if possible, unusually rich, and its illustrations profuse and of high artistic excellence. Personally, we think Harper's the best, as it most assuredly is the most readable of the magazines. Useful information is adroitly commingled with fiction and fun, so that the most dissipated reader *will* get some of the former, *nolens volens.*

The Magazine alone, $4 a year. The same each for the Weekly and Bazar. Either two $7, or the three $10. Draft or P. O. Order to Harper & Brothers, New York.

***College Courant—Yale.***

Charles C. Chatfield & Co, New Haven, Conn.; $4 a year. The *Courant* fully sustains the character we gave it last year. It is ably edited, and ranks among its contributors names of the highest distinction. The associations of college life are those which we last of all could "willingly let die." Wherever your *Alma Mater*, "Yale" is nevertheless no remote relative, and the *Courant* keeps itself and its readers well posted in the collegiate and general educational affairs and news of the country.

***The American Builder—***

A Journal of Art—Charles D. Lakey, Editor; Stanley Waterloo, Associate. $3 per annum; single copies 25 cents. The No. (for December) of this first class and now thoroughly established monthly exhibits, if possible, continued improvement. It is brimful of excellent matter pertaining to architecture, art, insurance and miscellany.

The subscription price also entitles each subscriber to a copy of the celebrated steel line engraving, size 24×32 inches, of Washington Irving. The editor owns the original plate, and copies cannot otherwise be procured. Any of our subscribers contemplating building, would find it greatly to their advantage to read this valuable publication, so that they can with some confidence speak of "what they know about building."

